# Endovascular Treatment for False Lumen Expansion Due to a Gutter Leak After Total Arch Replacement Using the Fenestrated Frozen Elephant Trunk Technique: A Case Report

**DOI:** 10.7759/cureus.109601

**Published:** 2026-05-25

**Authors:** Yusuke Shintani, Hiroyuki Otsuka, Shinya Negoto, Ryo Kanamoto, Seiji Onitsuka

**Affiliations:** 1 Division of Cardiovascular Surgery, Department of Surgery, Kurume University School of Medicine, Kurume, JPN

**Keywords:** aortic dissection, endovascular treatment, false lumen enlargement, frozen elephant trunk technique, gutter leak, total arch replacement

## Abstract

Acute aortic dissection is a life-threatening condition that requires urgent surgical intervention. The frozen elephant trunk (FET) technique, including the use of the J Graft Frozenix®, is an effective strategy for managing complex aortic pathologies. Approaches for left subclavian artery LSA reconstruction are a critical component of complex aortic arch procedures, such as total arch replacement or thoracic endovascular aortic repair (TEVAR), designed to prevent stroke, spinal cord ischemia, and left arm claudication. Approaches generally fall into the following three categories: in situ reconstruction (direct surgical reimplantation), extra-anatomical bypass, and endovascular techniques (e.g., chimney grafts, fenestration). Recently, the fenestrated FET technique, which creates a fenestration in the stent graft corresponding to the origin of the LSA to preserve anterograde blood flow, has demonstrated favorable outcomes. However, endoleak-related complications have been reported. In this report, we present a case of false lumen expansion caused by an endoleak following the application of the fenestrated FET technique for managing acute Stanford type A aortic dissection. This case involved a dissection extending to the root of the LSA. Postoperative contrast-enhanced computed tomography (CT) demonstrated blood flow into the false lumen. Follow-up contrast-enhanced CT performed every six months revealed lumen enlargement, prompting TEVAR and coil embolization. The postoperative course was uneventful. We performed TEVAR and coil embolization to treat endoleak-induced false lumen expansion following total aortic arch replacement using the fenestrated FET technique for acute type A aortic dissection. Although favorable outcomes have been widely reported for the fenestrated FET technique, this approach may be unsuitable for cases with entry points near the subclavian artery, necessitating careful patient selection and meticulous follow-up.

## Introduction

The frozen elephant trunk (FET) technique in conjunction with total arch replacement (TAR) has been increasingly employed as a surgical strategy for the management of acute Stanford type A aortic dissection (AAAD) across multiple centers. FET provides better postoperative remodeling in the distal aorta compared with the conventional elephant trunk (ET) technique [1]. One of the major technical challenges in TAR is the reconstruction of the supra-aortic vessels, especially the left subclavian artery (LSA), which is located deep within the operative field. LSA reconstruction approaches are a critical component of complex aortic arch procedures, such as TAR or thoracic endovascular aortic repair (TEVAR), designed to prevent stroke, spinal cord ischemia, and left arm claudication. Reconstruction approaches generally fall into three categories, namely, in situ reconstruction (direct surgical reimplantation), extra-anatomical bypass, and endovascular techniques (e.g., chimney grafts and fenestration). Recently, the fenestrated FET technique has been introduced. This technique preserves blood flow to the LSA without the need for its reconstruction by creating a fenestration in the stent graft at the level of the subclavian artery origin. This approach may reduce operative time and procedure-related complications [2]. However, complications such as endoleaks (ELs) have also been reported following the use of the fenestrated FET technique. ELs are defined as persistent blood flow outside the lumen of the stent graft but within the aneurysm sac after endovascular aortic repair. Here, we report a case in which false lumen enlargement occurred following the fenestrated FET technique, requiring subsequent endovascular intervention.

## Case presentation

A 72-year-old woman was transported to our hospital with the chief complaint of a sudden onset of sharp back pain. She exhibited no associated symptoms such as syncope, limb paralysis, or lower extremity ischemia. On arrival, she was alert. Her blood pressure was 180/92 mmHg without inter-arm difference, and her heart rate was 92 beats/minute. Transthoracic echocardiography showed no significant valvular disease, with only mild left ventricular hypertrophy. Laboratory tests revealed no hepatic or renal dysfunction. The patient was obese, with a height of 143 cm and a weight of 80 kg, and was receiving olmesartan 40 mg for hypertension treatment. The patient had no family history of aortic disease. Contrast-enhanced computed tomography (CECT) demonstrated AAAD characterized by a patent false lumen, with the primary entry tear located on the greater curvature of the aortic arch and a reentry tear extending to the origin of the LSA. An emergency TAR was successfully performed using the fenestrated FET technique through a median sternotomy.

In this case, the LSA was deeply situated within the chest cavity due to severe obesity, rendering in situ reconstruction technically challenging. In addition, the left axillary artery was located 7.5 cm beneath the skin surface, making anastomosis with a prosthetic graft difficult and precluding extra-anatomical reconstruction.

Cardiopulmonary bypass was performed using the right femoral artery and bicaval cannulation. After the bladder temperature dropped to 26°C, circulation was arrested, and the ascending aorta was transected; anterograde selective cerebral perfusion was initiated. A 2.5 × 12 cm Frozenix (Japan Lifeline, Tokyo, Japan) was inserted and deployed into the true lumen. The Frozenix stent graft was deployed to ensure that the stented portion always covered the origin of the LSA. The LSA orifice was identified intraoperatively by direct inspection both inside and outside of the stent graft. The portion of the graft material corresponding to the LSA orifice was grasped intraluminally using right-angle forceps, and a fenestration was created using Metzenbaum scissors. Care was taken to limit the fenestration diameter to less than 1 cm. Adequate backbleeding from the LSA through the fenestration confirmed patency. Until this case, the site at which a hole was created in the prosthetic graft at the root of the LSA had not been sutured and secured at our institution. No intraoperative imaging guidance was used for fenestration sizing or positioning. A four-branched J-graft (Japan Lifeline, Tokyo, Japan) was anastomosed to the distal side of the aorta with 4-0 polyvinylidene difluoride sutures using a felt strip. The unbranched portion of a 2.4 cm four-branched J-graft (Japan Lifeline) was anastomosed to the proximal side of the ascending aorta with 4-0 polyvinylidene difluoride sutures using a felt strip. After resuming circulation, reconstruction of the left common carotid and brachiocephalic arteries was performed. The aortic cross-clamping and total circulatory arrest times were 143 and 84 minutes, respectively.

The postoperative course was uneventful. Postoperative CECT revealed preserved anterograde blood flow to the LSA through the fenestration in the FET; however, ELs from the LSA fenestration into the false lumen of the aortic arch were observed (Figure 1A). EL was managed conservatively, and the patient was discharged three weeks postoperatively. Follow-up CECT was performed every six months, revealing false lumen enlargement from 3 mm postoperatively to 18 mm at 18 months (Figure 1B). Because of this progressive morphological change, secondary intervention was deemed necessary and undertaken 18 months after the initial treatment. This involved stent graft deployment in Zone 2, followed by coil embolization from the origin of the subclavian artery to achieve EL control. Preoperative magnetic resonance imaging confirmed sufficient collateral circulation of the LSA; therefore, subclavian artery bypass was not performed.

**Figure 1:**
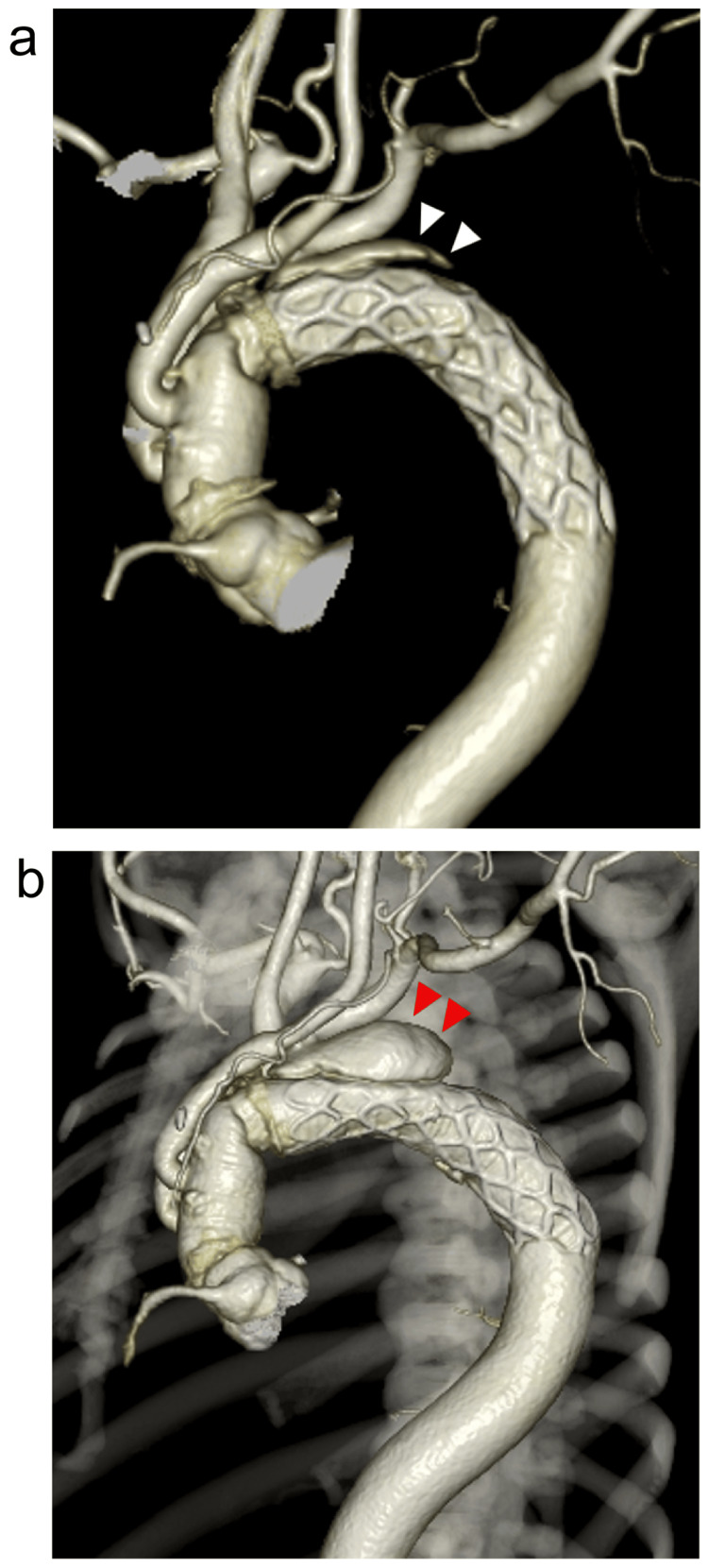
CECT findings. (a) CECT performed seven days after surgery using the fenestrated FET technique shows a small pseudocavity caused by an endoleak (white arrowheads). (b) CECT performed 18 months after surgery using the fenestrated FET technique shows marked enlargement of the false lumen with compression of the stented segment (red arrowheads). CECT: contrast-enhanced computed tomography; FET: frozen elephant trunk

Deployment of a stent graft (Valiant™ Thoracic Stent Graft with the Captiva™ Delivery System) in Zone 2 resulted in the resolution of the EL. Subsequently, a catheter was introduced via the left brachial artery, and coil embolization of the LSA was performed using microcoils (Figures 2A, 2B). Final angiography demonstrated complete resolution of contrast filling in the false lumen (Figure 2C). Postoperative CECT also confirmed the absence of ELs (Figure 3). Although a 30 mmHg blood pressure difference was observed between the upper limbs postoperatively, the patient exhibited no ischemic symptoms such as poikilothermia. The postoperative course was uneventful, and the patient was discharged on postoperative day seven.

**Figure 2:**
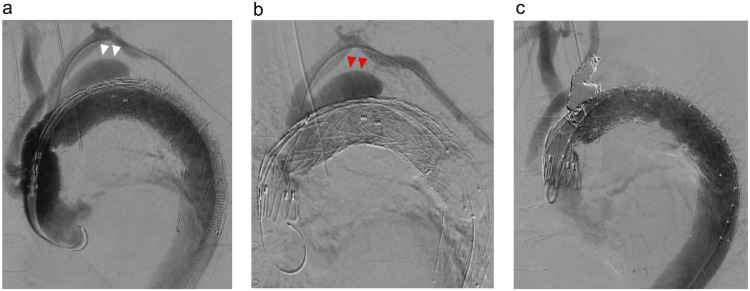
Angiography findings. (a) Angiography reveals a dilated false lumen (white arrowheads). (b) Persistent contrast enhancement of the false lumen was observed on angiography after TEVAR performed from Zone 2 (red arrowheads). (c) Angiography performed after coil embolization from the origin of the LSA demonstrated complete resolution of contrast filling in the false lumen. LSA: left subclavian artery; TEVAR: thoracic endovascular aortic repair

**Figure 3:**
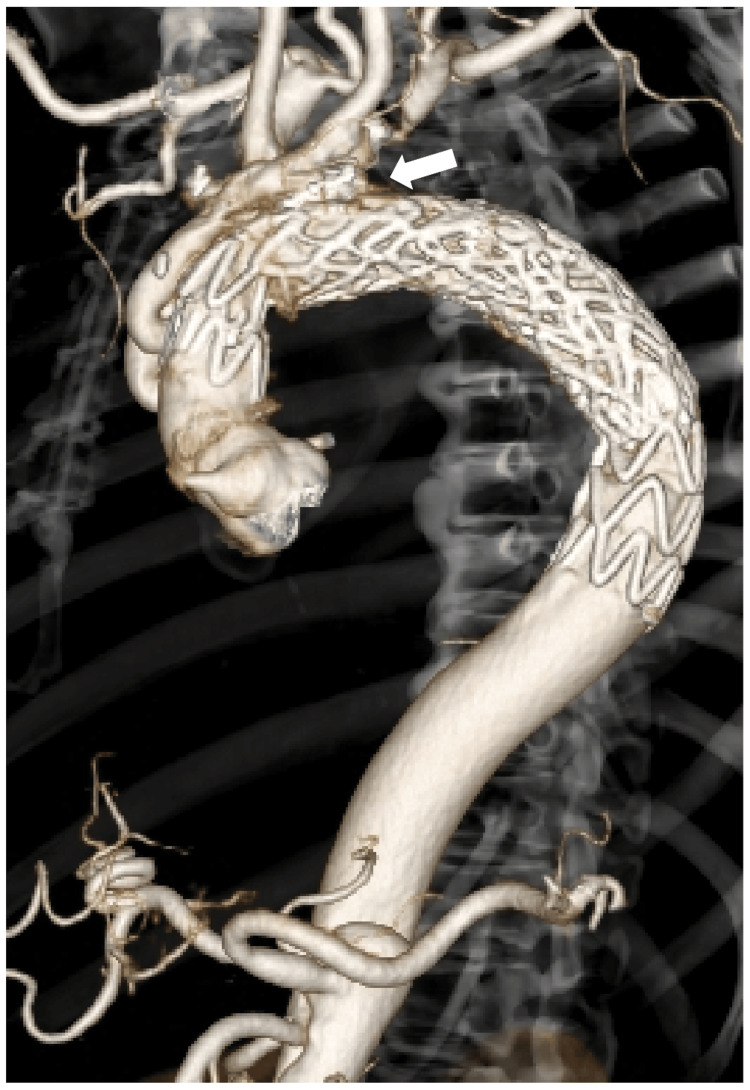
CECT findings. One week after surgery, CECT demonstrated complete absence of blood flow within the false lumen (white arrow). CECT: contrast-enhanced computed tomography

## Discussion

The introduction of stent implantation in aortic diseases in 1994 marked a new era. In 2003, the first attempt was made to integrate the ET technique into endovascular intervention. This innovative approach was developed by the Hannover group, led by Hans Georg Borst, the pioneer of the ET technique. He performed the procedure under hypothermic circulatory arrest, incorporating endovascular techniques. The added rigidity from the stent led Borst to name this new method “frozen ET” (FET), inspired by the stent’s “frozen” appearance on imaging. The concept of FET took an additional 20 years to fully evolve, culminating in the integration of stent implantation [3].

The fenestrated FET technique is an effective means of avoiding LSA anastomosis when performing TAR in cases of acute AAAD. In terms of surgical outcomes, TAR has been reported to reduce surgical and cardiopulmonary bypass times and yield perioperative mortality rates comparable to those of conventional surgery for acute AAADs. Compared with conventional TAR, the fenestrated FET technique has been associated with favorable five-year survival and aortic event-free survival rates in mid- to long-term follow-up. No ELs were observed regardless of whether the fenestrated section was fixed [4,5]. Okamura et al. reported that ELs from the fenestration area can be reliably controlled by suturing the fenestration area [4]. Azuma et al. did not suture the fenestration area but reported that ELs can be prevented by creating fenestrations of an appropriate size at appropriate locations within the stented portion. Furthermore, both teams ensured that the stent diameter did not exceed 110% to prevent gutter leaks caused by oversizing [5].

When performing TAR using the fenestrated FET technique, adhering to the exclusion criteria is important to prevent ELs. The fenestrated FET technique is not recommended in cases with dissection extending to the LSA origin, reentry within the LSA, a primary entry tear located on the greater curvature of the aortic arch, or distal arch dilatation [2,5]. In the present case, a reentry tear was located at the origin of the LSA, and thus the patient should have been considered ineligible for the procedure. In addition, the size of the fenestration created was 1 cm, which may have been excessive and could have contributed to the development of ELs [2]. Yamane et al. reported a case in which ELs from the fenestration led to expansion of the false lumen, causing compression of the true lumen and resulting in lower limb blood flow impairment. Although suture fixation of the fenestration was performed, the underlying cause was attributed to the fenestration created in the unstented artificial graft segment. Zone 2 TEVAR and subclavian artery coil embolization successfully closed the false lumen and improved the lower-limb blood flow [6]. In the present case, fenestration was created in the stented segment; nonetheless, suture fixation of the fenestration was not performed. Following this case, we adopted a strategy of fixing the proximal side of the fenestration with a single 4-0 monofilament suture using a pledget.

As an alternative method for preventing ELs, Rosselli et al. reported the placement of a stent within the fenestration, which demonstrated favorable outcomes [7]. Hashizume et al. reported that insertion of an open stent from Zone 1, combined with deployment of a branch vessel graft (5.0-cm-long Viabahn; Gore Medical) through the fenestration, obviates the need for cervical branch exposure and reduces operative time [8]. However, as Viabahn insertion for this indication is currently off-label, the development of dedicated devices will be necessary in the future.

## Conclusions

The introduction of the FET technique has simplified distal anastomosis, reduced operative time, and promoted distal aortic remodeling. Additionally, reliable reconstruction of the subclavian artery, tailored to each case, may help prevent cerebral infarction and spinal cord ischemia. In this case, we used a fenestrated FET technique to reconstruct the subclavian artery. However, we performed endovascular intervention to address false lumen enlargement caused by ELs from the fenestration following TAR using a fenestrated FET technique. Exclusion of cases with an entry tear near the LSA or a reentry tear within the LSA is essential for achieving favorable outcomes. Our case suggests possible reasons related to fenestration size (1 cm), lack of suture fixation, and entry tear location. However, causality has not been fully established, as the data is insufficient to definitively isolate the factor that caused the complication. Regular postoperative follow-up using CECT is important, as late false lumen enlargement has been reported.
